# “One of the Hardest Things I Have to Do in the Clinic”: A Survey of Veterinary Team Members’ Knowledge, Attitudes, and Practices Regarding Nail Clipping

**DOI:** 10.3390/vetsci13020115

**Published:** 2026-01-24

**Authors:** Anneshelly Chen, Evelyn Hall, Laura N. Bennington, Chantelle McGowan, Anne Quain

**Affiliations:** 1 Sydney School of Veterinary Science, University of Sydney, Camperdown, NSW 2006, Australia; 2 Department of Arts, Education and Agritech, Melbourne Polytechic, Epping, Melbourne, VIC 3076, Australia; 3 The Calm Pet Vet, Torrensville, Adelaide, SA 5031, Australia

**Keywords:** nail clipping, veterinary, nail trimming, dogs, cats, animal welfare, veterinary nurse, veterinary technician, fear-free, low-stress, animal handling, occupational safety

## Abstract

Nail clipping can cause fear, anxiety, and stress (FAS) in dogs and cats and possibly long-term aversion to veterinary care. We conducted an anonymous, online, mixed-methods survey to characterise the knowledge, attitudes, and practices of Australian veterinary nurses, technicians, and other veterinary staff regarding nail clipping in dogs and cats. Among 242 valid responses, 71.9% (*n* = 174/242) performed nail clipping multiple times per week. Dogs (72.4%, *n* = 165/228) and cats (59%, *n* = 134/227) frequently displayed FAS (score > 2/5) during nail clipping. Most respondents (79.8%, *n* = 193/242) reported being injured while performing nail clipping. Respondents reporting a negative attitude towards nail clipping were 5.5 times (95% CI = 1.7–17.8) more likely to report being injured during a nail clip compared to those with a positive attitude. Respondents reported feeling pressure to persevere with nail clipping when it was unnecessary or when animals exhibited severe FAS. Where used, pre-visit pharmaceuticals and sedation were reported to effectively reduce FAS (dog: *p* = 0.015; cat: *p* = 0.152), along with non-pharmaceutical interventions such as gentle handling and counter-conditioning. Respondents perceived nail-clipping to be a difficult, undervalued task. Increased training of veterinary team members regarding nail clipping could improve animal welfare and the safety of veterinary team members.

## Simple Summary

Nail clipping can cause fear, anxiety, and stress in dogs and cats, and possibly long-term aversion to veterinary care. This procedure is often performed by veterinary nurses, technicians, and other veterinary staff. We explored their knowledge, attitudes, and practices. Most veterinary team members clipped nails in dogs and cats multiple times per week. Most dogs and cats displayed fear, anxiety, and stress in association with nail clipping. Almost 4/5 of respondents had been injured during nail clipping. Those reporting a negative attitude towards nail clipping were more likely to report being injured during a nail clip compared to those with a positive attitude. Some felt pressure to persevere with nail clipping when they felt it was unnecessary or when animals exhibited severe distress. Where used, pre-visit pharmaceuticals and sedation effectively reduced fear, anxiety, and stress, along with non-pharmaceutical interventions such as gentle handling and counter-conditioning. In general, respondents felt that nail clipping was a difficult, undervalued task. Increased training of veterinary team members on this common yet difficult procedure has the potential to improve animal welfare and the safety of veterinary team members.

## 1. Introduction

Veterinary team members, including veterinary nurses, veterinary technicians, veterinarians, animal attendants, and other paraveterinary staff, are frequently requested to clip the nails of companion dogs and cats. A retrospective cross-sectional epidemiological study of dogs in the UK in 2019 reported that 5.6% of all dogs under veterinary care underwent at least one nail clip annually [1].

Appropriate nail length is important for maintaining normal gait and preventing injuries in dogs and cats [2]. Overgrown nails can cause discomfort, reduced mobility, and pain, especially if nails grow into the digital paw pads [3]. Differences in need for nail care may be due to variation in nail characteristics, such as quality, width, and hardness, or variation in animal characteristics, such as breed, conformation, age, health status, and activity levels [1]. For example, nail clipping becomes increasingly important in senior pets as degenerative joint disease may reduce the natural wear of nails. Older cats may be unable to retract overgrown nails, and long nails can become caught in fabric and other materials [4].

Many pet owners rely heavily on veterinary services to maintain their animals’ nails. Clients were almost equally likely to request nail clipping services from veterinary clinics and groomers [5]. Nail clipping was reported to be the primary reason for veterinary visits in 59.4% of cases where dogs’ nails were clipped, with the remainder of dogs undergoing nail clipping when presented for another reason (for example, vaccination) [1]. In this study, overgrown or ingrown nails (12.7%) and broken claws or dewclaws (8.8%) were the most common indications for nail clipping.

Yet for the animals themselves, nail clipping can be a source of fear, anxiety, and stress (FAS). Companion animals often experience FAS during veterinary visits [6]. These responses can be triggered by unfamiliar environments, direct or indirect exposure to other animals, physical restraint, handling by strangers, and separation from persons familiar to them [7]. The cumulative effect of these triggers is known as “trigger stacking” [8]. In addition to these triggers, nail clipping in itself may be an aversive experience. In a study where dogs underwent experimental nail clipping in a mock veterinary setting (*n* = 35), almost half showed signs of distress during the procedure. As only one nail per paw was clipped in this controlled setting, it is likely that clipping all of the nails (typically, 18 nails in most dogs and cats, though this may be increased in the presence of additional dew claws or polydactyly [9]) in clinical practice may exacerbate FAS [5]. Moreover, aversion and the active resistance to nail clipping may be associated with underlying musculoskeletal pain, for example, osteoarthritis [10].

Veterinary team members may also be negatively impacted. Fear, anxiety, and stress experienced by animals during nail clipping may lead to aggression. For example, in dogs and cats, FAS may manifest behaviourally as escape attempts, vocalisation, and aggression, including biting and scratching [7,11]. Fearful animals may scratch or bite in self-defence, while those handling animals that are struggling may experience knocks to the head if an animal swings its head, or strains or sprains in attempts to restrain animals. Scratches from dogs and cats can lead to injury and infection with zoonotic diseases or inoculation of saprophytic organisms. For example, cat scratches can transfer *Bartonella henselae* (cat scratch fever) [12], while scratches from dogs may be implicated in transmission of *Capnocytophaga canimorsus* [13], both of which can lead to severe, life-threatening disease in humans. In some countries, dog scratches have been implicated in the transmission of rabies [14]. One Australian study reported that 98% of veterinary nurses had been bitten or scratched by a dog or cat at work, with 11% contracting cat scratch fever [15].

In turn, negative experiences at the vet, including negative interactions with veterinary team members, can lead clients to delay or seek to minimise veterinary care for their animals. This was reflected in a survey of 2188 US dog and cat owners, where 40% of the participating cat owners did not bring their cats to the veterinarian due to experiencing a previous stressful visit [16]. In a survey investigating stress associated with veterinary visits in cats, 31.3% of cat owners (*n* = 82/262) reported that they had delayed a veterinary visit because they had previously witnessed stress in their cats during a veterinary visit [17]. As behaviours reinforced through emotional and physical trauma can be highly resistant to extinction, even a single negative experience can have a profound and lasting impact on an animal’s behaviour in veterinary settings [18,19]. Distress experienced by both animals and their owners during nail clipping may impose a barrier to regular nail care [20]. Concerningly, this may also be a barrier to veterinary care in general. There are concerns that routine veterinary care may lead to life-long negative impacts on animals, which highlights the need to refine veterinary care to minimise patient FAS [21].

Nail clipping is predominantly performed by veterinary nurses, technicians, or other paraveterinary staff, yet the literature is “veterinarian-centric” [22]. Despite the frequency of this procedure, there is currently no published research investigating the knowledge, attitudes, and practices of veterinary team members performing nail clipping. The primary objective of this study was to characterise the knowledge, attitudes, and practices of veterinary team members performing nail clipping in dogs and cats in Australian veterinary practices. A secondary objective was to identify potential refinements to improve the welfare of animals undergoing nail clipping.

## 2. Materials and Methods

### 2.1. Survey Instrument

This cross-sectional study utilised an anonymous, online, mixed-methods survey. The survey, developed specifically for this study, was built on RedCAP (Research Electronic Data Capture), a secure, web-based platform hosted by the University of Sydney. Survey questions were developed based on a review of the literature, and in discussion with all authors (one qualified veterinary nurse, one veterinary student working as a veterinary nurse, one veterinarian with Membership of the Australian and New Zealand College of Veterinary Scientists (ANZCVS) Behaviour Chapter and one veterinarian working in primary care who is also a Diplomate of the European College of Animal Welfare and Behaviour Medicine (ECAWBM) in Animal Welfare Science, Ethics and Law (AWSEL)). The survey referred to the Spectrum of Fear, Anxiety, and Stress scoring systems for dogs and cats [23] commonly displayed in veterinary hospitals around the world, including in Australia, asking respondents to rate FAS according to a five-point scale. Surveys were piloted with two small animal veterinarians, one veterinary behaviour consultant, and two registered veterinary nurses. Feedback that improved the clarity of questions was incorporated into the final version.

The survey consisted of closed (binary and drop-down responses) and open-ended questions. For drop-down and multi-answer questions, if prefilled responses did not include the respondent’s most preferred option(s), an option labelled “other” was made available for applicable questions, allowing respondents to provide free-text responses.

The survey comprised 4 sections: (1) demographic questions; (2) general questions regarding nail clipping in small animals; (3) questions specific to nail clipping in canine patients; and (4) questions specific to nail clipping in feline patients (questions and options for responses are provided in full in Supplementary Table S1).

### 2.2. Recruitment, Consent, and Ethics Approval

The link to the survey was distributed through the Veterinary Nurses Council of Australia (VNCA) via their online newsletter and through the VNCA Facebook page. The survey was open to veterinary nurses, veterinary technicians, paraveterinary staff, and other veterinary team members [24,25,26,27,28] who undertook nail clipping. Participants were required to be 18 years old or over and currently working in Australia. The survey was open from March 2025 to June 2025. Participation in this survey was completely voluntary with no incentives offered. The survey was approved by The University of Sydney Human Research Ethics Committee (project number 2024/HE001319). The participant information statement was the landing page of the survey, and consent was indicated by pressing the submit button at the end of the survey.

### 2.3. Data Cleaning

Survey data were downloaded from REDCap into Microsoft**^®^** Excel**^®^** (Version 16.98, Build 25060824). Only responses from those who clicked the “submit” button, indicating consent to participate, were retained. Where applicable, “other” (free-text) responses describing a category that was already listed in the drop-down menu of potential responses in the survey were recategorised into that pre-existing category deleted from the “other” category. Responses were retained and denoted as “other” if the free-text responses could not be recategorised. Respondents who selected “other” but did not provide a free-text response were excluded from the “other” category.

### 2.4. Descriptive Analysis

Descriptive analyses were performed for all categorical variables. IBM SPSS Statistics 29 was used to facilitate the descriptive analysis.

### 2.5. Statistical Analysis

All statistical analyses were conducted using Jamovi (v2.6.26). A *p*-value of <0.05 was considered significant. Data were categorised into three individual sets: general (all data), dogs, and cats. Outcomes included attitudes of the veterinary staff towards nail clipping (positive, neutral, and negative), injury to the person clipping the nails (yes/no), and patient fear scale (fear/no fear). To facilitate logistic regression, the fear scale was categorised into a binary outcome: displaying fear or not displaying fear (0 if respondents answered that the animal was displaying fear scale 0 or 1; 1 if respondents answered that the animal was displaying fear scale 2, 3, 4, or 5). Explanatory variables included years of experience (<1 yr, 1–3 yrs, 4–5 yrs, 6–10 yrs, 11+ yrs), certification in Fear Free**^®^**, Low Stress Handling**^®^**, Stress Free Pets**^®^** or ISFM accreditation (completed/not completed), location performing nail clipping (common treatment area, consult room with owner, consult room without owner, other), nail clipping training (none, theory, practical, on job), attitude towards nail clipping, other procedures in conjunction with nail clipping (yes/no) and the use of pre-visit pharmaceuticals (PVPs) (not given, given and effective, given but ineffective). For binary outcomes, a logistic regression with an underlying binomial distribution was used to assess any associations between the outcome and explanatory variables. An ordinal logistic regression analysis was used to determine any effect of the explanatory variables on the attitude outcome. *p* values, odds ratios (OR), and their 95% confidence intervals (CI) along with predicted proportions and their standard errors (se) were reported.

### 2.6. Coding of Responses to Open-Ended Questions

For responses to open-ended questions (excluding those describing treats offered in association with nail clipping), a codebook thematic analysis was used [29]. Responses were imported into Microsoft Word and read multiple times by two authors (AQ and AC) to familiarise themselves with the data. These files were imported into NVivo to facilitate analysis. A codebook was developed for responses to each open-ended, free-text question. Where responses reflected distinct themes, these could be coded multiple times. Coding was an iterative process. Codes were reviewed for internal coherence and distinctiveness from other codes. Codes that were not distinct were combined. At completion of coding, tables containing a list of codes, frequencies of coding, and excerpts from coded abstracts were constructed. We have previously used this approach to analyse free-text responses to veterinary surveys [30].

Free-text responses describing treats fed to dogs or cats were analysed using the “Word Frequency” query function in NVivo to determine the most frequent treats fed. Words under three letters, or those that described the patient (“dog” or “cat”), type of treat (such as “dried”), generic words (such as “treat” or “treats”) were excluded.

## 3. Results

### 3.1. Descriptive Analysis

A total of 246 responses were received; however, four respondents failed to press the “submit” button to indicate consent, and were thus excluded. Therefore, 242 valid survey responses were analysed. The distribution of categorical demographic variables is described in Table 1.

All respondents had clipped at least one dog or cat’s nails in the previous 12 months, with the majority clipping nails in both species. Most respondents (94.2%) were female, and most (74.0%) were qualified veterinary nurses (see Table 1). Of those who selected “other”, 11 were veterinarians, and one worked as a veterinary assistant.

Among the respondents, 77.3% had at least 4 years of experience.

Most respondents worked within metropolitan (49.2%) and regional areas (43.4%), with just 5.4% and 2.1% working in rural and remote areas, respectively. Around two-thirds (66.5%) worked primarily at small animal practices that provided general or mobile services (private and corporate-owned). Of the three respondents who selected “other”, one worked in a veterinary nursing education organisation, one worked in both shelter and private practice, and one worked at TAFE (Technical and Further Education, a vocational educational and training provider).

Most respondents had completed various types of stress-reducing animal handling accreditation (69.8%) (Table 2). Of the three respondents who selected “other”, one stated they had completed a non-animal-related course, one had completed a certification offered by a pharmaceutical company, and one person reported watching webinars on Fear Free^®^ care but did not report obtaining a certification.

The majority of respondents reported a neutral attitude towards nail clipping (52.5%), with approximately one quarter reporting disliking nail clipping (24.8%), compared with less than one-fifth (18.2%) reporting liking nail clipping. Few respondents actively sought opportunities to perform nail clipping.

Respondents most frequently reported clipping nails of conscious patients at least weekly, with 41.3% clipping nails multiple times per week, compared with multiple times a day (19%) and daily (11.6%).

Most respondents had received on-the-job training regarding nail clipping (66.1%). Almost one in five (19.8%) received no training or were self-taught.

Most respondents (79.8%) reported that they had been injured when performing nail clipping.

Respondents most frequently performed nail clipping in an area of the practice accessible to staff only (68.2%), followed by a consult room with the owner present (23.6%). Of the four people who selected “other location within the practice”, one used both a consult room with the owner present and the common treatment area equally, two determined the location based on the patient, and one person performed nail clipping at a training institution (TAFE).

Most respondents (78.5%) reported that they sometimes took animals out of the owner’s sight for nail clipping. The most frequent reasons for doing so were to seek help from more staff (78.5%), because animals were easier to handle when their guardians were not present (70.2%), to perform nail clipping in a more appropriate area (42.1%), and the owner did not want to see (31.8%). Only 9.1% reported that they did so because they did not want owners to see. Of the 11 respondents who selected “other”, four respondents reported that nail clipping was performed in hospitalised patients. Of the remaining respondents, one wrote that patients requiring nail clips were brought to them from the consultation room by the veterinarian, one wrote that it was up to the owners if they were present or absent, one wrote that there were no consult rooms available due to the size of the clinic and the time of bookings, one wrote that unless owners requested nail clipping as part of another procedure it was always performed during consults with the owner present, one wrote that removing animals from the owner’s sight during nail clipping was practice policy as it helped animals behave, one wrote that it facilitated appropriate safety and handling, and one wrote that they did so to be nearer equipment such as topical anticoagulant gel/powder to manage potential complications such as bleeding.

The most frequently reported factor causing animals the most stress in association with nail clips was pain or discomfort around nails/toes (34.7%), followed by physical restraint (21.5%), loss of control (16.8%), and the location where the nail clips were performed (7.4%). Of the 43 respondents (17.8%) who selected “other”, responses were lack of training/desensitisation with paws being handled (*n* = 13), animals generally do not like their paws being touched (*n* = 12), fear/trauma/anticipation of pain caused by previous experience of nail clips (*n* = 11), a combination of multiple factors listed in the survey (*n* = 9), noise generated during nail clips (*n* = 7), and general stress associated with vet visit/being in an unfamiliar environment (*n* = 5).

#### 3.1.1. Dogs

Most respondents (94.2%) had clipped a dog’s nails in the previous 12 months. For the last dog nail clip that the respondents performed, most were performed during visits where dogs underwent additional procedures (62.2%), while the nail clip was the sole reason for the visit in 37.7% of cases (see Table 3).

Almost all (92.5%) respondents gave treats to dogs when performing nail clips. The treats most frequently offered to dogs were liver (*n* = 165), chicken (*n* = 40), cheese (*n* = 31), and peanut butter (*n* = 24).

Most respondents reported that two people were usually in physical contact with the dog during a nail clip (75%).

More than 2/3 of the respondents answered that PVPs were not administered prior to the visit in the last dog whose nails they clipped (75.9%). Out of the dogs that received PVPs (20.6%), 57.4% reported that PVPs were effective, and 42.6% reported that they were ineffective.

In just over one-fifth of cases (22.4%), respondents reported that some bleeding occurred in the last dog whose nails they clipped.

**Figure 1:**
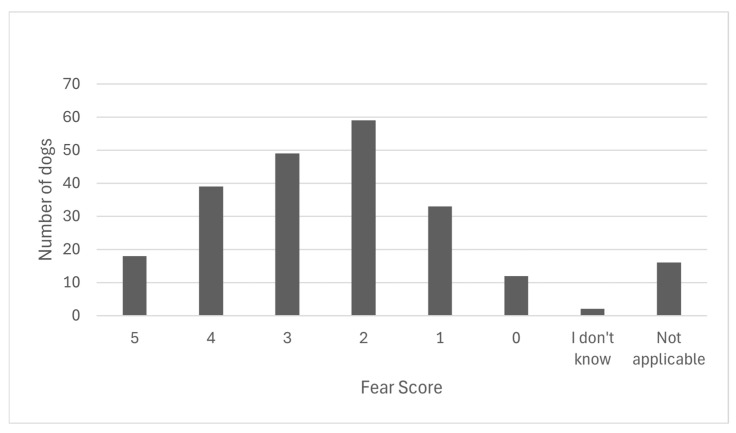
The distribution of fear scores, on a scale of 0–5, adapted from the Fear Free Spectrum of Fear, Anxiety, and Stress [23], reported by Australian veterinary nurses, technicians, and other veterinary team members regarding the dog whose nails they most recently clipped within the last 12 months.

In the last dog nail clip that the respondents performed, 7.9% reported that the dog was displaying a scale 5 of FAS, 17.1% rated a scale 4 FAS in their patients, 21.5% reported a scale 3 FAS, 25.9% reported a scale 2 FAS, 14.5% reported a scale 1 FAS, and 5.3% reported a scale 0 FAS. There were 0.9% that did not know the FAS rating of their patients, and 7% selected that it was not applicable in their patients (for example, if the patient was anaesthetised for another procedure) (see Figure 1).

Respondents most frequently selected giving treats/ad hoc counter conditioning (65.8%), PVPs (64.0%), and desensitisation (57.0%) as the most useful methods of reducing stress in canine patients.

#### 3.1.2. Cats

Most respondents (93.8%) had clipped a cat’s nails in the last 12 months (Table 4). For the last cat nail clip that the respondents performed, more than half (52.9%) were performed during visits where cats underwent additional procedures, while the nail clip was the sole reason for the visit in 47.1% of cases.

Over half of respondents reported that they give treats to cats when performing nail clips (59.9%). However, 40.1% of respondents did not give treats at all when performing nail clips. The treats most frequently offered to cats were different brands of squeezable puree tubes (*n* = 132) and commercial dental treats (*n* = 32).

The majority reported that two people were usually in physical contact with the cat during a nail clip (80.6%).

Most respondents reported that cats were not given PVPs prior to the visit (85%). Regarding the 34 cats who received PVPs, 82.4% reported that PVPs were effective, and 17.6% reported that they were ineffective.

**Figure 2:**
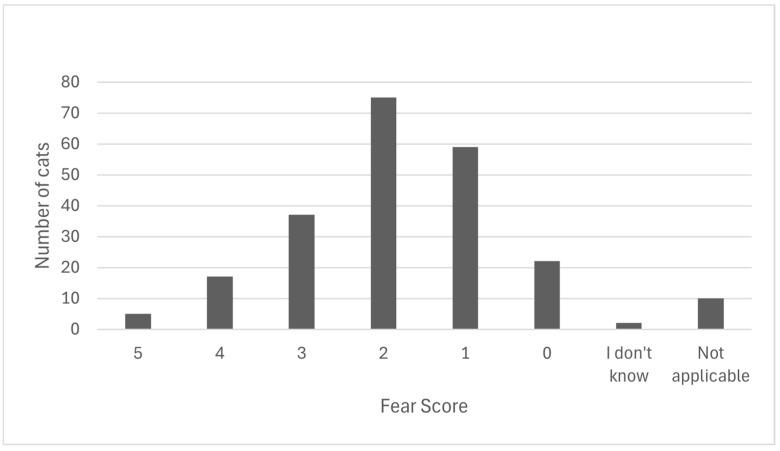
The distribution of fear scores, on a scale of 0–5, adapted from the Fear Free Spectrum of Fear, Anxiety, and Stress [23], reported by by Australian veterinary nurses, technicians, and other veterinary team members regarding the cat whose nails they most recently clipped within the last 12 months. *Respondents most frequently selected PVPs (47.1%), giving treats/ad hoc counter conditioning (37.9%), and desensitisation (29.5%) as the most useful methods of reducing stress in feline patients*.

In the last cat nail clip that the respondents performed, 2.2% reported that the cat displayed a scale 5 FAS, 7.5% rated a scale 4 FAS in their patients, 16.3% reported a scale 3 FAS, 33% reported a scale 2 FAS, 26% reported a scale 1 FAS, and 9.7% reported a scale 0 FAS. A minority (0.9%) did not know the FAS rating of their patients, while 4.4% selected that it was not applicable in their patients (see Figure 2).

### 3.2. Statistical Analysis

Results of the statistical analysis are reported in Tables S2–S6. There was a significant association between having been injured during a nail clip and the attitude of the person performing nail clipping (*p*-value = 0.003) (see Table S2). Respondents reporting a negative attitude towards nail clipping were 5.5 times (95% CI = 1.7–17.8) more likely to report having been injured during a nail clip compared to those reporting a positive attitude.

There was a significant association between displaying signs of fear and having nail clipping performed alone in dogs (*p*-value = 0.036) (see Table S4). Dogs were 2.1 times more likely to show fear if they only received a nail clip than if they received a nail clip in conjunction with other procedures.

In dogs, a significant association was found between displaying signs of fear and the use of PVPs (*p*-value = 0.015) (see Table S6). Dogs not given PVPs were 2.9 times (95% CI 1.1–7.4) more likely to show fear compared to dogs for whom PVPs were reported to be effective. In contrast, dogs for whom PVPs were not deemed effective were 12.7 times (95% CI 1.4–14.4) more likely to show fear than dogs for whom PVPs were effective. No significant associations between parameters explored (for example, displaying signs of fear and having nail clipping performed alone or in conjunction with other procedures) were found in cats, possibly reflecting the smaller size of the feline subgroup.

### 3.3. Codebook Thematic Analysis

In response to the question regarding alternative approaches suggested where a dog’s nails could not be clipped, there were 189 free-text responses (2532 words). The most frequently coded themes were “Sedation or general anaesthesia to facilitate nail clipping” (*n* = 87), “Dispense pre visit pharmaceuticals” (*n* = 69), “Desensitisation and or counter-conditioning at home” (*n* = 27), “Recommend use of a scratch board or scratch pad at home to wear down nails” (*n* = 24), and “Recommend wearing nails down on rough surfaces by walking on concrete or similar” (*n* = 17) (see Table S7).

In response to the question regarding alternative approaches suggested where a cat’s nails could not be clipped, there were 129 free-text responses (1038 words). The most frequently coded themes were “Sedation or general anaesthesia to facilitate nail clipping” (*n* = 68), “Dispense pre visit pharmaceuticals” (*n* = 42), “Postpone nail clip until the animal is having sedation or a general anaesthetic for another reason” (*n* = 13), “Recommend the use of scratching posts or scratch pads at home to wear nails down” (*n* = 11), “Desensitisation and or counter-conditioning at home” (*n* = 8), and “Recommend owner clips or files nails at home” (*n* = 8) (see Table S8).

There were 238 responses to the question regarding how a respondent might approach clipping an animal’s nails if resources were not limited, comprising 7093 words. The most frequent themes were “Desensitisation and counter-conditioning at the vet (including use of treats, toys)” (*n* = 119), “Nail clips should be performed under sedation general anaesthetic or with PVPs on board more frequently or always” (*n* = 110), “Resources to educate owners regarding nail management at home” (*n* = 83), “More time to approach animal and nail clipping slowly” (*n* = 71) and “Clip nails in a quiet calm environment or dedicated space where animals are most comfortable” (*n* = 51) (see Table S9). Of the 238 responses, only 5 indicated that they felt there was nothing about the way they were performing nail clipping that could be improved.

There were 68 respondents wishing to add a final comment, comprising 3544 words. The most frequent themes were “Nail clips are undervalued by clients or veterinary team members” (*n* = 21), “There is a need for more training of veterinary team members regarding nail clipping and minimising patient fear, anxiety, and stress” (*n* = 19), “We should not be performing nail clipping as frequently, or at all, in veterinary settings” (*n* = 14), “Approaches for minimising fear anxiety and stress during nail clipping” (*n* = 14), “There is pressure to get the job done” (*n* = 9) and “There should be more time to take breaks, clip nails incrementally and to take individual animal needs into account” (*n* = 9) (see Table S10).

## 4. Discussion

Nail clipping was very common in Australian veterinary practices, with most respondents performing this procedure at least multiple times per week. Respondents reported high rates of FAS in animals undergoing nail clipping, with 72.4% of dogs and 59% of cats displaying an FAS score of 2/5 or above. This finding aligns with literature showing that veterinary visits can be a source of FAS for dogs and cats [11]. We suspect that respondents underestimated FAS. An experimental, cross-sectional study analysing FAS scoring with the Fear Free Spectrum of Fear, Anxiety, and Stress (FFSFAS) in dogs visiting veterinary practices (*n* = 79) reported that while the instrument had good concurrent validity and intra-rater reliability for most participants, all participants (animal owners, veterinarians, and behavioural experts) displayed high percentages (50.9–64.9%) of incorrect scores, with mid-range FAS categories particularly challenging to score [31]. That study did not include veterinary nurses. The authors argued that high rates of incorrect scores suggest a need for both refinement of the instrument and dedicated training of staff using the instrument. In our study, less than half of the respondents had completed a handling certification and may not have been familiar with FAS scoring using the FFSFAS. It is likely that fewer were trained to use it. The VNCA guidelines specify that veterinary nurses should be competent in recognising negative impact on animal welfare when handling or restraint exceeds their tolerance level and should report patient pain to veterinarians [32]. However, the guidelines do not require reporting FAS to veterinarians.

Concerningly, 79.8% of respondents reported being injured when performing a nail clip, such as bites, scratches, head knocks, sprains, and strains. While we did not ask about the nature and severity of injuries incurred during nail clipping, previous studies of injuries to veterinary personnel suggest that these may include animal bites or scratches, which may lead to damage to joints and tendons, infections, sickness, absenteeism, and chronic injury [33]. A previous survey of Australian veterinarians (*n* = 494), veterinary nurses (*n* = 484), and veterinary students (*n* = 212) reported that 33%, 44%, and 23%, respectively, had experienced a cat bite in the previous year [33]. It is unknown if any of these injuries occurred during nail clipping. A retrospective analysis of workers’ compensation claims among animal care workers (including veterinary support staff) in the United States (US) found that animal-related trauma, including bites, kicks, and scratches, was the most commonly reported injury type associated with all veterinary patients [34]. Furthermore, support staff had higher claim counts than veterinarians. While we found a significant association between having been injured during nail clipping and having a negative attitude towards nail clipping, we are unable to determine whether being injured predisposed a negative attitude toward nail clipping or a negative attitude toward nail clipping predisposed to injury. Pushing animals beyond their limits may increase the risk of being bitten or scratched. It may also transgress the values of veterinary team members, leading to moral distress around feeling complicit in harming animals [35]. Witnessing animal distress has been shown to cause emotional strain among veterinary support staff [36]. Moral distress and moral injury can cause long-term psychological distress in veterinary professionals, such as guilt, shame, anger, and loss of confidence [37]. Employers have a duty of care to keep persons in the workplace physically and psychologically safe [38]. While this study did not assess mental health or moral distress in respondents, this could be explored in future studies.

Some respondents reported feeling pressure from owners, employers, or both to persevere with nail clipping even where animals became very stressed and to complete the task as quickly as possible. Some reported refusal of veterinarians to recognise the need for PVPs or sedation. These factors may increase the risk of injury. It is vital, for the safety and welfare of patients and veterinary team members, that all veterinary team members feel empowered to raise concerns [39,40].

These findings underscore the need to refine nail clipping to promote the wellbeing of veterinary patients and veterinary team members. A key consideration is whether nails need to be clipped in the first place. This was raised in free-text responses, with some respondents feeling pressure from clients or employers to clip nails that did not require clipping. Keeping nails short and less abrasive may reduce risks to owners and may be particularly important to prevent injury in those with health conditions or taking medications that may suppress the immune system or increase the risk of haemorrhage. Nail clipping may also reduce physical discomfort of persons when interacting with companion animals [41]. However, these concerns need to be balanced with animal welfare considerations. Apart from the potential for FAS, respondents reported that one or more nails bled (suggesting that the quick had been cut) in just over one-fifth of dogs undergoing nail clipping.

Together with the risk of human injury, we argue that the decision of whether to clip nails should be considered on a case-by-case basis rather than being performed routinely. There are no guidelines describing normal or optimal nail length [1], making it difficult for veterinary team members to convince a client that nail clipping is not needed. In free-text comments, several respondents expressed frustration regarding requests for nail clips they felt were unnecessary or where they perceived that the benefits of clipping were outweighed by the costs (notably stress to the animal and veterinary team members involved). Having brought their animal to the vet, owners may be reluctant to leave without having had the nails clipped. Similarly, veterinary practices may be reluctant to withhold a requested service for fear of disappointing a client and forgoing income. The pressure imposed by clients or employers to persevere with the procedure may increase the risk of a painful and aversive experience for animals and possibly anticipation of pain in association with future veterinary visits. Animals may demonstrate FAS when their paws are handled, if not previously desensitised. Those introduced to nail clipping at an older age can show more pronounced fear during veterinary visits when compared with younger animals [42].

**Figure 3:**
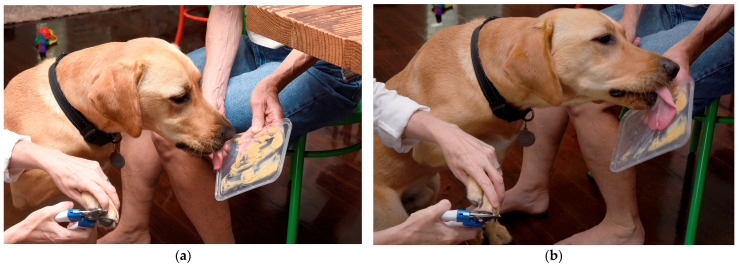
An example of counter-conditioning in a two-year-old, male-neutered Labrador who has previously exhibited anxiety in veterinary settings, having his nails clipped at home. (**a**) shows the commencement of nail clipping. One member of the household offers a high-value reward (peanut butter smeared on the lid of a plastic container), which the dog licks off as another member of the household clips his nails. (**b**) shows the dog progressively licking the peanut butter as the nail clip continues. Counter-conditioning involves the pairing of a potentially aversive experience (paw handling and nail clipping) with a highly desirable reward (in this case, peanut butter) to reinforce calm behaviour, known as counter-conditioning. Note that the value of the reinforcer depends on the individual animal. Note that if peanut butter is used it should be free of artificial sweeteners like xylitol as these can be toxic to dogs. Photographs taken by Michael Quain and reproduced with permission.

Where nail clipping is required, steps can be taken to reduce FAS. Non-pharmacological strategies reported to reduce FAS in veterinary patients include minimal restraint, ad hoc counter-conditioning with treats or toys, environmental management such as starting a consult outside the clinic where safe and appropriate, and preventative strategies such as “happy visits” where animals are rewarded with treats when visiting veterinary clinics, but no examination or treatment is involved [22]. The use of desensitisation, counter-conditioning, acclimation, and the availability of a quiet and appropriate space were selected by the respondents as the most useful non-pharmaceutical stress-reducing techniques. A challenge in Australian clinics, highlighted by some respondents, is a lack of consult rooms available. Respondents called for longer appointment times to allow for nail clipping and ensuring an adequate number of staff are available to assist as required [6,7,43]. As suggested by several respondents, another strategy to reduce FAS associated with nail clipping in suitable cases is nail clipping or filing at home by the owner. This can be combined with counter-conditioning, as in Figure 3a,b.

Behavioural distress negatively impacts patient welfare and potentially patient health, veterinary care, and client adherence to care plans [44]. While non-pharmacological strategies to address behavioural distress due to FAS are preferred, pharmacological intervention is often required to reduce acute situational FAS to avoid long-term harm to patient welfare and improve personnel safety [44]. Pharmacological strategies were used by a minority of respondents, but where they were used, they were generally reported to be effective. Many respondents expressed that PVPs, sedation, or even general anaesthesia should be used more frequently than currently to facilitate nail clipping. A survey of dog owners from the US (*n* = 513) reported that 59.5% were aware of the availability of PVPs to alleviate FAS in dogs. Barriers to use included concerns regarding psychoactive pharmaceuticals in animals, causing profound sedation or the perceived risk of the animal becoming addicted [45]. However, a high proportion of dog owners (77.4%) reported that a recommendation from a veterinarian was one of the most important factors when considering the use of pharmaceuticals for FAS.

As many respondents commented, general anaesthesia for any reason (for example, neutering, dentistry, or other surgical procedures) presents an opportunity to clip the patient’s nails while they are unconscious, thereby avoiding FAS associated with nail clipping. Rather than asking each client whether they would like an animal’s nails clipped while they are anaesthetised, the use of an “opt-out” consent may ensure that the maximum number of veterinary patients benefit from this procedure.

Most respondents relied on on-the-job training for nail clipping, and almost one-fifth received no training. Given both the impacts on animal welfare and the high risk of injury associated with nail clipping, we echo the call of Ahmed and colleagues for increased training of veterinary team members regarding nail clipping [1]. Additionally, there is a need for more training of veterinary team members in scoring and documenting FAS for animals. It is important that all veterinary team members, including those not directly handling animals, are appropriately trained. This ensures that they can work closely together to identify and mitigate risks. A survey of small animal practices in the US and Canada (*n* = 113) found that practices with less than 100 percent of employees certified in stress-reducing patient care programs were 3.5 times more likely to report injuries once per month or more compared to those with 100 percent employee certification [46]. Practices where all employees were certified reported increased use of PVPs, pheromone spray, highly palatable food, and use of towels and blankets for animal handling, with decreased use of muzzles, cat nets, and “rabies poles” to handle fearful animals. Certification of the entire veterinary team may remove barriers to implementing FAS-reducing animal care and handling. A 2023 survey of Australian veterinary professionals working with dogs (*n* = 291) found that just one-fifth reported having a stress-reducing certification [22]. Certified professionals were significantly more likely to agree that their work environment was set up to facilitate stress-reducing veterinary care and to practice general and specific strategies in stress-reducing veterinary care than veterinary professionals without a certification. Given the frequency of nail clipping and the FAS in dogs and cats that may occur, it is crucial to identify the most effective stress-reducing technique for individual patients.

Our findings align with previous research exploring barriers to the implementation of stress-reducing care in dogs in Australian practices, including workplace and practice management, workload, colleagues, clients, clinic environment, work-specific conflict, personal abilities, and patients [22]. The authors reported that “implementing strategies to improve patient welfare as part of routine practice likely still requires significant changes in workplace and industry culture”, a statement also supported by our findings.

For the safety and comfort of patients and veterinary team members involved, it is important to ensure that appropriate, functioning equipment is available. Several respondents raised the need for better or appropriate equipment to perform nail clipping, echoing the recommendations of Ahmed and colleagues [1].

Overall, we found that nail clipping is a frequent procedure, the risks of which are often underappreciated. Furthermore, as indicated by minimal specific training, the skills required to perform nail clipping are underestimated. Appropriate time and resource allocation are required to perform nail clipping in a way that minimises harm and optimises patient welfare and wellbeing for dogs, cats, and veterinary team members. As this procedure is often carried out by veterinary nurses and paraveterinary staff, we recommend training of all veterinary team members.

### Limitations and Future Directions

A key limitation of this study is the lack of data relating to the population sampled, precluding the calculation of response rates. Recruitment was focused on but not limited to members of the VNCA, as VNCA social media channels are followed by members and non-members, posts could be on-shared, and the VNCA e-newsletter is sent to current members, past members, and broader industry contacts. Care is needed in generalising these results to non-VNCA members or those who do not engage with the VNCA. There is no available data on the overall number of veterinary nurses, animal health technicians, practice managers, and paraveterinary staff currently employed in Australia. Another limitation is the small sample size. For example, the number of veterinary nurses employed in Australia was projected to grow from 15,400 to 17,800 from 2021 to 2026 [47]. Assuming an average, the current number is 16,600 (the average between these figures); the 198 respondents who identified as qualified or unqualified veterinary nurses represent 1.2% of the total population. The offer of an incentive may have increased response rates, but as incentives are also associated with an increased risk of non-genuine participation in online research, we chose not to incentivise participation [48]. Nonetheless, veterinary nurses, technicians, animal attendants, and other paraveterinary staff are an understudied population when compared with veterinarians. Furthermore, this is the first study to document the knowledge, attitude, and practice of these veterinary team members who are primarily responsible for nail clipping in dogs and cats. Due to the low number of respondents working in rural and remote areas, a cautious approach should be taken in generalising these findings, as caseload, staffing, and economic factors may vary substantially from metropolitan and regional areas [49]. However, the smaller proportion of respondents in rural and remote workplaces reflects the distribution of veterinary services in Australia [50].

One of the challenges of voluntary surveys is non-response bias, as the findings only represent the knowledge, attitudes, and practices of those who were willing to participate in the survey [51]. This may have attracted respondents with stronger feelings regarding nail clipping. Alternatively, potential respondents with strong negative feelings about nail clipping may have found it stressful to recall their experiences and therefore declined to participate in the study. Similarly, surveys may be impacted by social desirability bias, whereby respondents answer according to what they believe researchers desire. We minimised social desirability bias by ensuring complete anonymity. Recall bias is a limitation in retrospective surveys [51]. We sought to minimise this by asking respondents about the most recent dog and/or cat nail clip they had performed in the previous 12 months.

Based on our own experience working in veterinary practices in Australia and in piloting the survey, we assumed that respondents understood that PVPs are potentially anxiolytic and/or sedative agents administered by the client to their animal prior to the veterinary visit, while sedation is administered by a veterinarian during a visit. Although the survey distinguished between PVPs and sedation, it is possible that some respondents viewed “sedation” as the administration of any agent with a sedative effect prior to or during the visit. This may have impacted their responses.

Given the frequency of nail clipping and potential for negative impacts on both patients and veterinary team members, it would be useful to develop and pilot a training program, informed by an understanding of how animals learn and stress-reducing principles, to educate owners about nail care of animals in the home, where appropriate and safe to do so. Such a program could be supported by veterinary professional organisations such as the VNCA and the Australian Veterinary Association (AVA).

Longitudinal studies are required to determine the impact of specific training and the use of both pharmaceutical and non-pharmaceutical stress-reducing measures on both FAS scores of animals having their nails clipped and injury rates of veterinary team members performing this procedure.

## 5. Conclusions

Nail clipping is commonly performed in Australian veterinary clinics, with potential negative impacts on animals and personnel. Most dogs and cats having their nails clipped displayed signs of FAS, and most respondents reported having been injured during nail clipping, underscoring a need to refine this procedure. It is important to correctly identify the FAS scores of the animal, as severe FAS may indicate and cause harm to both the patient and the person performing nail clipping. Refinement of this procedure may start with training the veterinary professional on how animals learn and stress-reducing handling techniques. This study also highlights the need to explore strategies to address barriers to the use of both pharmaceutical and non-pharmaceutical stress-reducing measures. Finally, there is a need to train all veterinary team members in nail clipping using techniques to minimise fear, anxiety, and stress in animals.

## Figures and Tables

**Table 1:** Categorical demographic information from a survey of Australian veterinary nurses, technicians, and other veterinary team members who performed nail clipping of a dog or cat within the last 12 months (*n* = 242).

Demographic Parameters	Category	Number	Percentage (%)
What is your gender?	Female	228/242	94.2%
	Male	10/242	4.1%
	Non-binary	4/242	1.7%
How many years of experience do you have in clinical practice?	Less than a year	7/242	2.9%
	1–3 years	48/242	19.8%
	4–5 years	55/242	22.7%
	More than 6 years but less than 10 years	57/242	23.6%
	More than 11 years	75/242	31%
What best describes your role in clinical veterinary practice?	Qualified veterinary nurse	179/242	74%
	Veterinary nurse without a qualification	19/242	7.9%
	Other	11/242	4.5%
	Licenced veterinary technician	10/242	4.1%
	Animal attendant/Kennel hand	8/242	3.3%
	Trainee/Student	7/242	2.9%
	Veterinary technician without a licence	5/242	2.1%
	Volunteer	2/242	0.8%
	Veterinary receptionist	1/242	0.4%
What is the type of your primary workplace?	Private small animal practice—general or mobile	108/242	44.6%
	Corporate-owned private small animal practice—general or mobile	53/242	21.9%
	Private small animal practice—referral or emergency	23/242	9.5%
	Private mixed animal practice	18/242	7.4%
	Corporate-owned small animal practice—referral or emergency	17/242	7.0%
	Animal shelter/Non-profit organisation/Charity	10/242	4.1%
	Corporate-owned mixed animal practice	5/242	2.1%
	University teaching hospital	5/242	2.1%
	Other	3/242	1.2%
Where is your workplace located?	Metropolitan—major capital cities	119/242	49.2%
	Regional areas—includes towns, small cities, and areas that lie beyond the major capital cities	105/242	43.4%
	Rural—sits outside a regional centre but is within a few hours’ drive	13/242	5.4%
	Remote—a township far removed from a major capital or regional centre	5/242	2.1%

**Table 2:** Training, frequency, and experience of nail clipping in dogs and cats in a survey of Australian veterinary nurses, technicians, and other veterinary team members who performed nail clipping of a dog or cat within the last 12 months (*n* = 242).

General Parameters	Category	Number	Percentage (%)
Have you completed any Fear Free^®^/Low Stress^®^/Stress Free^®^/ISFM^®^ accreditation?	Fear Free^®^	89/242	36.8%
	Stress Free Pets^®^	45/242	18.6%
	ISFM (International Society of Feline Medicine) Cat Friendly Clinic Accreditation^®^	20/242	8.3%
	Low Stress Handling^®^	15/242	6.2%
	No, but I plan to complete it	59/242	24.4%
	No, I have not completed it	54/242	22.3%
	Other	3/242	1.2%
How do you feel about performing nail clips?	I actively seek them out	10/242	4.1%
	I enjoy them	44/242	18.2%
	I do not have positive or negative feelings	127/242	52.5%
	I dislike them	60/242	24.8%
	I refuse to perform them	1/242	0.4%
How often do you typically perform nail clips in a conscious patient?	Less than once a month	30/242	12.4%
	Less than once a week	38/242	15.7%
	Multiple times per week	100/242	41.3%
	Daily	28/242	11.6%
	Multiple times a day	46/242	19.0%
Which of the following best fits the training you have received specifically on nail clips?	Practical—on-the-job training	160/242	66.1%
	Theoretical training (part of a course, e.g., Fear Free, ACVN, TAFE, etc)	71/242	29.3%
	Practical—part of a certificate course	54/242	22.3%
	None/Self-taught	48/242	19.8%
Have you ever been injured during the process of nail clipping? This may include bites, scratches, head knocks, sprains, strains, etc.	Yes	193/242	79.8%
	No	49/242	20.2%
Where do you most commonly perform nail clips?	Area of the practice accessible to staff only (e.g., common treatment area)	165/242	68.2%
	Consult room with owner present	57/242	23.6%
	Consult room without owner present	14/242	5.8%
	Other	4/242	1.7%
	Home visit	2/242	0.8%
	Outside of the clinic building (e.g., car park, garden)	0/242	0.0%
	Waiting room	0/242	0.0%
Do you take animals out of the owner’s sight for nail clipping?	Never	7/242	2.9%
	Sometimes	190/242	78.5%
	Always	45/242	18.6%
What were the reasons that the animal was taken out of the owner’s sight?	To seek help from more staff	190/242	78.5%
	Animals are easier to handle when owners are not present	170/242	70.2%
	To perform nail trims in a more appropriate area	102/242	42.1%
	Owners did not want to see	77/242	31.8%
	Did not want owners to see	22/242	9.1%
	Other	11/242	4.5%
In your opinion, what do you believe causes animals the most stress in association with nail clipping?	Pain or discomfort around nails/toes	84/242	34.7%
	Physical restraint	52/242	21.5%
	Other	43/242	17.8%
	Loss of control	41/242	16.9%
	Location (e.g., in clinic, car park, home, etc.)	18/242	7.4%
	Separation from the owner	4/242	1.7%

**Table 3:** Frequency and experiences of nail clipping in dogs by Australian veterinary nurses, technicians, and other veterinary team members within the last 12 months (*n* = 242).

Dog Parameters	Category	Number	Percentage (%)
Have you clipped a dog’s nails in the last 12 months?	Yes	228/242	94.2%
	No	14/242	5.8%
For the last nail clip in a dog, what other procedures were performed in the same consult/visit?	None—nail clip only	86/228	37.7%
	Physical examination	93/228	40.8%
	Injection (e.g., vaccination, heartworm injection, monoclonal antibody)	87/228	38.2%
	Anal gland expression/rectal exam	60/228	26.3%
	Blood draw	41/228	18.0%
	Dental check	28/228	12.3%
	Other	25/228	11.0%
	Ear examination/otoscopy	22/228	9.6%
	Clipping fur	22/228	9.6%
	Eye examination/ophthalmoscopy	16/228	7.0%
	FNA—fine needle aspiration	11/228	4.8%
	Suture removal	11/228	4.8%
	Cystocentesis	6/228	2.6%
	Free-catch urine collection	6/228	2.6%
For dogs, do you give treats during nail clipping?	Yes—before	146/228	64.0%
	Yes—during	195/228	85.5%
	Yes—after	176/228	77.2%
	No	17/228	7.5%
How many people (including the owner, if involved) are typically in physical contact with the dog during a nail clip? (e.g., someone holding an animal, someone clipping nails, and someone giving treats = 3 people)	1	5/228	2.2%
	2	171/228	75.0%
	3	52/228	22.8%
	4 or more	0/228	0%
For the last dog whose nails you clipped, were pre-visit pharmaceuticals administered prior to the visit?	No	173/228	75.9%
	Yes, and it was effective	27/47	57.4%
	Yes, but it was ineffective	20/47	42.6%
	I do not know	8/228	3.5%
For the last dog whose nails you clipped, was sedation administered in the clinic?	No	203/228	89.0%
	Yes, and it was effective	22/25	88%
	Yes, but it was ineffective	3/25	12%
	I do not know	0/228	0%
For the last dog whose nails you clipped, did any of the nails bleed?	No	177/228	77.6%
	Yes	51/228	22.4%
On a scale of 0–5 (0 being not fearful, anxious, or stressed, and 5 including intense displays of fight/flight/freeze/fiddle/fawn responses), rate the stress level of the last dog whose nails you clipped.	0	12/228	5.3%
	1	33/228	14.5%
	2	59/228	25.9%
	3	49/228	21.5%
	4	39/228	17.1%
	5	18/228	7.9%
	I do not know	2/228	0.9%
	Not applicable	16/228	7.0%
Has there ever been a dog you have not been able to clip the nails of while conscious (even if sedated or given pre-visit pharmaceuticals)?	Yes	197/228	86.4%
	No	31/228	13.6%
For the last dog nail clip you can recall performing, which of the following have you found the most useful in reducing stress?	Giving treats/ad hoc counter conditioning (i.e., counter conditioning without desensitisation), including lickmats, kongs, etc	150/228	65.8%
	Pre-visit pharmaceuticals	146/228	64.0%
	Desensitisation (teaching owners how to desensitise at home)	130/228	57.0%
	Sedation	77/228	33.8%
	Head tapping	68/228	29.8%
	Other distractions	46/228	20.2%
	Firm restraint	35/228	15.4%
	Muzzle	35/228	15.4%
	Toys/ball	7/228	3.1%
	Not applicable	5/228	2.2%

**Table 4:** Frequency and experiences of nail clipping in cats by Australian veterinary nurses, technicians, and other veterinary team members within the last 12 months (*n* = 242).

Cat Parameters	Category	Number	Percentage (%)
Have you clipped a cat’s nails in the last 12 months?	Yes	227/242	93.8%
	No	15/242	6.2%
For the last nail clip in a cat, what other procedures were performed in the same consult/visit?	None—nail clip only	107/227	47.1%
	Physical examination	88/227	38.8%
	Injection (e.g., vaccination, heartworm injection, monoclonal antibody)	59/227	26.0%
	Blood draw	32/227	14.1%
	Dental check	21/227	9.3%
	Clipping fur	17/227	7.5%
	Other	17/227	7.5%
	Ear examination/otoscopy	10/227	4.4%
	Eye examination/ophthalmoscopy	8/227	3.5%
	Cystocentesis	5/227	2.2%
	Suture removal	5/227	2.2%
	FNA—fine needle aspiration	4/227	1.8%
	Free-catch urine collection	3/227	1.3%
	Anal gland expression/rectal exam	2/227	0.9%
For cats, do you give treats during nail clipping?	Yes—before	71/227	31.3%
	Yes—during	116/227	51.1%
	Yes—after	100/227	44.1%
	No	91/227	40.1%
How many people (including the owner, if involved) are typically in physical contact with the cat during a nail clip? (e.g., someone holding an animal, someone clipping nails, and someone giving treats = 3 people)	1	26/227	11.5%
	2	183/227	80.6%
	3	18/227	7.9%
	4 or more	0/227	0.0%
For the last cat whose nails you clipped, were pre-visit pharmaceuticals given prior to the visit?	No	193/227	85.0%
	Yes, and it was effective	28/34	82.3%
	Yes, but it was ineffective	6/34	17.6%
For the last cat whose nails you clipped, was sedation administered in the clinic?	Yes, and it was effective	17/17	100%
	No	210/227	92.5%
	Yes, but it was ineffective	0/17	0.0%
On a scale of 0–5 (0 being not fearful, anxious, or stressed, and 5 including intense display of fight/flight/freeze/fiddle/fawn responses), rate the stress level of the last cat whose nails you clipped.	0	22/227	9.7%
	1	59/227	26.0%
	2	75/227	33.0%
	3	37/227	16.3%
	4	17/227	7.5%
	5	5/227	2.2%
	I do not know	2/227	0.9%
	Not applicable	10/227	4.4%
Has there ever been a cat you have not been able to clip the nails of while conscious (even if sedated or given pre-visit pharmaceuticals)?	Yes	141/227	62.1%
	No	86/227	37.9%
For the last cat nail clip you can recall performing, which of the following have you found the most useful in reducing stress?	Pre-visit pharmaceuticals	107/227	47.1%
	Towel wrap/cat bag	93/227	41.0%
	Giving treats/ad hoc counter conditioning (i.e., counter conditioning without desensitisation), including lickmats, kongs, etc	86/227	37.9%
	Desensitisation (teaching owners how to desensitise at home)	67/227	29.5%
	Sedation	63/227	27.8%
	Other distractions	30/227	13.2%
	Firm restraint	28/227	12.3%
	Head tapping	24/227	10.6%
	Not applicable	20/227	8.8%
	Toys/ball	4/227	1.8%
	Muzzle	2/227	0.9%

## Data Availability

The data presented in this study are available on request from the corresponding author due to restrictions imposed by the ethics committee.

## Supplementary Materials

The following supporting information can be downloaded at https://www.mdpi.com/article/10.3390/vetsci13020115/s1, Table S1: Survey questions; Table S2: *p*-values for assessing association between injury and attitude and other variables; Table S3: Predicted values, odd ratios and 95% confidence intervals for factors associated with injury during a nail clip; Table S4: *p*-values for fear scale analyses in dogs and cats; Table S5: Predicted values, odd ratios and 95% confidence intervals for factors associated with cat fear scale and procedures at the vet; Table S6: Predicted values, odd ratios and 95% confidence intervals for factors associated with dog fear scale and procedures at the vet; Table S7: Codes and frequencies for free text responses to the question “Has there ever been a dog you have not been able to clip the nails of while conscious (even if sedated or given pre-visit pharmaceuticals)?”, “If yes, did you suggest an alternative approach?” (*n* = 189); Table S8: Codes and frequencies for free text responses to the question “Has there ever been a cat you have not been able to clip the nails of while conscious (even if sedated or given pre-visit pharmaceuticals)?”, “If yes, did you suggest an alternative approach?” (*n* = 129); Table S9: Codes and frequencies for free text responses to the question “If resources were not limited, how might you approach clipping an animal’s nails?” (*n* = 238); Table S10: Codes and frequencies for free text responses to the question “Is there anything else you want to add?” (*n* = 68).

## Abbreviations

The following abbreviations are used in this manuscript:

ACVN	Australian College of Veterinary Nursing
ANZCVS	Australian and New Zealand College of Veterinary Scientists
AVA	Australian Veterinary Association
AWSEL	Animal Welfare Science, Ethics, and Law
ECAWBM	European College of Animal Welfare and Behaviour Medicine
FAS	Fear, anxiety, stress
FNA	Fine needle aspirate
PVP	Pre-visit pharmaceuticals
TAFE	Technical And Further Education
US	United States
VNCA	Veterinary Nurses Council of Australia
