# Supplementary material for: “One of the Hardest Things I Have to Do in the Clinic”: A Survey of Veterinary Team Members’ Knowledge, Attitudes, and Practices Regarding Nail Clipping
Source: Vet Sci. 2026 Jan 24;13(2):115. doi: 10.3390/vetsci13020115 (PMC12945167; doi:10.3390/vetsci13020115)
Supplement: Supplementary file 1 [file vetsci-13-00115-s001.zip › vetsci-4097426-supplementary.pdf]

Supplementary Information.

Please note we have intentionally left this page largely blank to ensure the questions in the remainder of the table are not split across pages.

Table S1: Survey questions

I have read the Participant Information Statement.	◯ Yes<br>◯ No<br>(Answer yes to consent to participate, and no if you do not wish to participate.)
Are you 18 years old of age or older?	◯ Yes<br>◯ No<br>(Respondents must be 18 years of age or older to participate in this study.)

What best describes your role in clinical veterinary practice?*[See Key below]	◯ Qualified veterinary nurse<br>◯ Licensed veterinary technician<br>◯ Veterinary nurse without a qualification<br>◯ Veterinary technician without a license<br>◯ Veterinary receptionist<br>◯ Animal attendant/Kennel hand<br>◯ Trainee/Student<br>◯ Volunteer<br>◯ Other<br>(Select one option only)
If other, please specify.	______________________________<br>(If your answer is not in the drop down menu, please specify)
How many years of experience do you have in clinical practice?	◯ Less than a year<br>◯ 1-3 years<br>◯ 4-5 years<br>◯ More than 6 years but less than 10 years<br>◯ More than 11 years<br>(Select one option only)
What is your gender?	◯ Female<br>◯ Male<br>◯ Non-binary<br>◯ Other - please specify<br>(Select one option only)
If other, please specify.	______________________________<br>(If your answer is not in the drop down menu, please specify)
What is the type of your primary workplace?	◯ Private small animal practice - general or mobile<br>◯ Cooperate-owned private small animal practice - general or mobile<br>◯ Private small animal practice - referral or emergency<br>◯ Cooperate-owned small animal practice - referral or emergency<br>◯ Private mixed animal practice<br>◯ Cooperate-owned mixed animal practice<br>◯ University teaching hospital<br>◯ Animal shelter/Non-profit organisation/Charity<br>◯ Other - please specify<br>(Select one option only)
If other, please specify.	______________________________<br>(If your answer is not in the drop down menu, please specify)

Where is your workplace located?	◯ Metropolitan - major capital cities<br>◯ Regional areas - includes all of the towns, small cities and areas that lie beyond the major capital cities<br>◯ Rural - sits outside a regional centre, but is within a few hours' drive<br>◯ Remote - a township far removed from a major capital or regional centre<br>(Select one option only)
Have you completed any Fear Free®/Low Stress®/Stress Free®/ISFM® accreditation?	☐ Fear Free®<br>☐ Low Stress®<br>☐ Stress Free®<br>☐ ISFM® - International Society of Feline Medicine<br>☐ No, I have not done it<br>☐ No, but I plan to do it<br>☐ Other - please specify<br>(Select all that apply)
If other, please specify.	______________________________<br>(If your answer is not in the drop down menu, please specify)
How do you feel about performing nail clips?	◯ I actively seek them out<br>◯ I enjoy them<br>◯ I don't have positive or negative feelings<br>◯ I dislike them<br>◯ I refuse to perform them<br>(Select one option only)
Why? Any specific experience that makes you feel that way?	______________________________
How often do you typically perform nail clips in a conscious patient?	◯ Less than once a month<br>◯ Less than once a week<br>◯ Multiple times per week<br>◯ Daily<br>◯ Multiple times a day<br>(Select one option only)
Which of the following best fits the training you have received specifically on nail clips?	☐ None/Self-taught<br>☐ Theoretical training (part of a course e.g. Fear Free, ACVN, TAFE, etc)<br>☐ Practical - part of a certificate course<br>☐ Practical - on the job training<br>(Select one option only)
Have you ever been injured during the process of nail clipping? This may including bites, scratches, head knocks, sprains, strains, etc.	◯ Yes<br>◯ No<br>(Select yes or no)

Where do you most commonly perform nail clips?	◯ Outside of the clinic building (e.g. car park, garden)<br>◯ Waiting room<br>◯ Consult room with owner present<br>◯ Consult room without owner present<br>◯ Area of the practice accessible to staff only (e.g. common treatment area)<br>◯ Home visit<br>◯ Other<br>(Select one response only)
If other, please specify.	__________________________________<br>(If your answer is not in the drop down menu, please specify)
Do you take animals out of the owner's sight for nail clipping?	◯ Never<br>◯ Sometimes<br>◯ Always<br>(Select one option only)
What were the reasons that the animal was taken out of the owner's sight?	☐ To seek help from more staff<br>☐ To perform nail trims in a more appropriate area<br>☐ Did not want owners to see<br>☐ Owners did not want to see<br>☐ Animals are easier to handle when owners not present<br>☐ Other - please specify<br>(Select all that apply)
If other, please specify.	__________________________________<br>(If your answer is not in the drop down menu, please specify)

The following questions are about nail clipping in dogs. If you have not clipped dog nails in the last 12 months, you will be directed to the questions relating to cats once you select the answer no.

Have you clipped a dog's nails in the last 12 months?	◯ Yes<br>◯ No<br>(If you select no, you will be directed to the cat section of the survey)

For the last nail clip in a dog, what other procedures were performed in the same consult/visit?	☐ None - nail clip only<br>☐ Physical examination<br>☐ Blood draw<br>☐ Injection (e.g. vaccination, heartworm injection, monoclonal antibody)<br>☐ Anal gland expression/rectal exam<br>☐ Dental check<br>☐ Ear examination/otoscopy<br>☐ Eye examination/ophthalmoscopy<br>☐ FNA - fine needle aspiration<br>☐ Cystocentesis<br>☐ Free-catch urine collection<br>☐ Clipping fur<br>☐ Suture removal<br>☐ Other<br>(Select all that apply)
If other, please specify.	_________________________<br>(If your performed procedure was other than listed above please specify)
For dogs, do you give treats during nail clips?	☐ Yes - before<br>☐ Yes - during<br>☐ Yes - after<br>☐ No<br>(Select all that apply)
If yes, what kind of treats do you give to dogs?	_________________________<br>(If you select yes, please specify the treats you give to dogs)
How many people (including the owner, if involved) are typically in physical contact with the dog during a nail clip? (e.g. someone holding an animal, someone clipping nails and someone giving treats = 3 people)	○ 1<br>○ 2<br>○ 3<br>○ 4 or more<br>(Select one option only)
For the last dog whose nails you clipped, were pre-visit pharmaceuticals administered prior to the visit?	○ Yes, and they were effective<br>○ Yes, but they were ineffective<br>○ No<br>○ I don't know<br>(Select one option only)
For the last dog whose nails you clipped, was sedation administered in the clinic?	○ Yes, and it was effective<br>○ Yes, but it was ineffective<br>○ No<br>○ I don't know<br>(Select one option only)
For the last dog whose nails you clipped, did any of the nails bleed?	○ Yes<br>○ No<br>(Select yes or no)
If yes, how did you stop the bleeding? (e.g. compression bandage, styptic gel/powder, etc.)	_________________________<br>(Please describe the method you used to stop the bleeding)

On a scale of 0-5 (0 being not fearful, anxious, or stressed, and 5 including intense displays of fight/flight/freeze/fiddle/fawn responses), rate the stress level of the last dog whose nails you clipped.

◯ 0
◯ 1
◯ 2
◯ 3
◯ 4
◯ 5
◯ I don't know
◯ Not applicable - animal adequately sedated/anaesthetized
(Select one option only)

Has there ever been a dog you have not been able to clip the nails of while conscious (even if sedated or given pre-visit pharmaceuticals)?

◯ Yes
◯ No
(Select yes or no)

If yes, did you suggest an alternative approach?

____
(If you suggested an alternative approach to the owner, please specify)

For the last dog nail clip you can recall performing, which of the following have you found the most useful in reducing stress?

☐ Pre-visit pharmaceuticals
☐ Sedation
☐ Desensitisation (teaching owners how to desensitise at home)
☐ Giving treats/Ad hoc counter conditioning (i.e. counter conditioning without desensitisation) including lickmats, kongs etc
☐ Toys/ball
☐ Other distractions
☐ Head tapping
☐ Firm restraint
☐ Muzzle
☐ Not applicable
(Select all that apply)

The following questions are about nail clipping in cats.

Have you clipped a cat's nails in the last 12 months?

◯ Yes
◯ No
(If you select no, you will be directed to the last section of the survey)

For the last nail clip in a cat, what other procedures were performed in the same consult/visit?

☐ None - nail clip only
☐ Physical examination
☐ Blood draw
☐ Injection (e.g. vaccination, heartworm injection, monoclonal antibody)
☐ Anal gland expression/rectal exam
☐ Dental check
☐ Ear examination/otoscopy
☐ Eye examination/ophthalmoscopy
☐ FNA - fine needle aspirate
☐ Cystocentesis
☐ Free-catch urine collection
☐ Clipping fur
☐ Suture removal
☐ Other - please specify
(Select all that apply)

If other, please specify.	______________________________<br>(If your answer is not in the drop down menu, please specify)
For cats, do you give treats during nail clips?	☐ Yes - before<br>☐ Yes - during<br>☐ Yes - after<br>☐ No<br>(Select all that apply)
If yes, what kind of treats do you give to cats?	______________________________<br>(If you select yes, please specify the treats you give to cats)
How many people (including the owner, if involved) are typically in physical contact with the cat during a nail clip? (e.g. someone holding an animal, someone clipping nails and someone giving treats = 3 people)	◯ 1<br>◯ 2<br>◯ 3<br>◯ 4 or more<br>(Select one option only)
For the last cat whose nails you clipped, were pre-visit pharmaceuticals given prior to the visit?	◯ Yes, and they were effective<br>◯ Yes, but they were ineffective<br>◯ No<br>(Select one option only)
For the last cat whose nails you clipped, was sedation administered in the clinic?	◯ Yes, and it was effective<br>◯ Yes, but it was ineffective<br>◯ No<br>(Select one option only)
On a scale of 0-5 (0 being not fearful, anxious, or stressed, and 5 including intense display of fight/flight/freeze/fiddle/fawn responses), rate the stress level of the last cat whose nails you clipped.	◯ 0<br>◯ 1<br>◯ 2<br>◯ 3<br>◯ 4<br>◯ 5<br>◯ I don't know<br>◯ Not applicable - animal adequately sedated/anaesthetized<br>(Select one option only)
Has there ever been a cat you have not been able to clip the nails of while conscious (even if sedated or given pre-visit pharmaceuticals)?	◯ Yes<br>◯ No<br>(Select yes or no)
If yes, did you suggest an alternative approach?	______________________________<br>(If you select yes, did you suggest an alternative approach to the owner?)

For the last cat nail clip you can recall performing, which of the following have you found the most useful in reducing stress?

- ☐ Pre-visit pharmaceuticals
- ☐ Sedation
- ☐ Desensitisation (teaching owners how to desensitise at home)
- ☐ Giving treats/Ad hoc counter conditioning (i.e. counter conditioning without desensitisation) including lick mats, kongs etc
- ☐ Toys/mice
- ☐ Other distractions
- ☐ Head tapping
- ☐ Firm restraint
- ☐ Towel wrapping / cat bag
- ☐ Muzzle
- ☐ Not applicable

(Select all that apply)

This is the last section of the survey.

In your opinion, what do you believe causes animals the most stress in association with nail clips?

- ◯ Separation from the owner
- ◯ Physical restraint
- ◯ Pain or discomfort around nails/toes
- ◯ Loss of control
- ◯ Location (e.g. in clinic, carpark, home, etc.)
- ◯ Other - please specify

(Select one option only)

If other, please specify.

____
(If your answer is not in the drop down menu, please specify)

If resources were not limited, how might you approach clipping an animal's nails?

____
(Resources might include time, equipment, staff, space or anything else)

Is there anything else you would like to add?

(Is there anything about the topic of nail clipping that we haven't covered or you wish to expand on)

***Key to roles described in the survey as they pertain to the Australian context.**

Qualified veterinary nurse	A person working as a veterinary nurse that holds a Certificate IV in Animal Care and Management Training Package (ACM40112), diploma or degree in veterinary nursing from an accredited training body[1, 2]. Registration of veterinary nurses is currently not required under Australian legislation, but the Veterinary Nurses Council of Australia launched the Australian Veterinary Nurse and Technologist (AVNAT) Registration Scheme on 1 April 2019[3].
Veterinary nurse without a qualification	A person working as a veterinary nurse who has not undertaken formal training and does not hold a vet nursing qualification from an accredited training body. Currently, outside of a single state (Western Australia), there is no regulation or statutory code of conduct, and no requirement to maintain currency and fitness to practice within veterinary nursing, however, national bodies including the Veterinary Nurses Council of Australia and Australian Veterinary Association agree that "unqualified and unregulated veterinary support staff potentially expose the public and animal patients to harm, and increase liability for veterinary practices"[4].
Licensed veterinary technician	A person working as a veterinary technician/technologist that has completed a bachelor's degree in veterinary technology from an accredited training body and passed state or national examinations and licensed in jurisdiction outside of Australia or is registered with the Veterinary Nurses Council of Australia[5]. Currently, many veterinary technologists working in Australia qualified overseas, however, there are now degree programs in veterinary technology offered in Australia. Registration of veterinary nurses is currently not required under Australian legislation, but the Veterinary Nurses Council of Australia launched the Australian Veterinary Nurse and Technologist (AVNAT) Registration Scheme on 1 April 2019[3].
Veterinary technician without a licence	A person working as a veterinary technician/technologist that passed state or national examinations and licensed in jurisdiction outside of Australia or is registered with the Veterinary Nurses Council of Australia[5].

Animal attendant/kennel hand	A person who works alongside veterinary nurses/technicians and veterinarians in a hospital/clinic setting as an assistant, whose main duties would include cleaning, restocking and basic husbandry and animal handling under direction and supervision.
Trainee/student	A person who is currently enrolled as a student in a formal qualification and either employed by or undertaking a training placement at a veterinary hospital/clinic.
Volunteer	A person who engages in unpaid volunteer work at a veterinary hospital/clinic.
Veterinary receptionist	A person whose role is reception and administrative duties at a veterinary hospital/clinic such as greeting clients, managing calls, scheduling and checking in appointments, handling payments, correspondence management and may perform basic animal handling
Other	A person who does not fit the above definitions, but has another role (as specified in their free-text responses) which may involve interacting with animals in a veterinary hospital/clinic.

Supporting references for key

1. Australian Veterinary Association. *Regulation of animal health service providers*. 2018 7 December 2018 [cited 2026 17 January, 2026].
2. Veterinary Nurse's Council of Australia. *What is a veterinary nurse?* 2026 [cited 2026 17 January 2026]; Available from: https://www.vnca.asn.au/about-veterinary-nursing/what-is-a-veterinary-nurse/#:~:text=The%20VNCA%20defines%20a%20%E2%80%9CVeterinary,within%20the%20veterinary%20nursing%20profession.
3. Veterinary Nurse's Council of Australia. *AVNAT Registration Scheme*. 2026 [cited 2026 17 January]; Available from: Registration of veterinary nurses is currently not required under Australian legislation, but the Veterinary Nurses Council of Australia launched the Australian Veterinary Nurse and Technologist (AVNAT) Registration Scheme on 1 April 2019.
4. Australian Veterinary Association. *Veterinary Nursing*. 2020 [cited 2026 17 January]; Available from: https://www.ava.com.au/policy-advocacy/policies/accreditation-and-employment-of-veterinarians/veterinary-nursing/.
5. Veterinary Nurse's Council of Australia. *For Vet Nurses and Vet Technologists*. 2026 [cited 2026 17 January, 2026]; Available from: https://www.vnca.asn.au/avnat-registration-scheme-/vet-nurses-and-vet-technicians/.

Table S2. P-values for assessing association between injury and attitude and other variables.

	**Injury (Y/N)**	**Attitude (+ve/neutral/-ve)**
Experience	0.066	0.919
Course	0.210	0.322
Location	0.465	-
Training (none)	0.370	-
Training (theory)	0.053	-
Training (practical course)	0.434	-
Training practical on job	0.142	-
Attitude	**0.003**	

Table S3. Predicted values, odd ratios and 95% confidence intervals for factors associated with injury

during a nail clip.

		proportion Yes	se	Odds Ratio	LCI	UCI	P value
Experience	<1yr	0.429	0.187	-			
	1-3yrs	0.729	0.064	3.59	0.71	18.26	
	4-5yrs	0.873	0.045	9.14	1.68	49.75	0.066
	6-10 yrs	0.842	0.048	7.11	1.36	37.31	
	11+yrs	0.787	0.047	4.92	0.997	24.25	
Course	Completed	0.763	0.039	-			0.210
	Not done	0.828	0.033	1.50	0.79	2.81	
Attitude	Positive	0.722	0.061	-			
	Neutral	0.764	0.038	1.24	0.604	2.56	0.003
	Negative	0.934	0.032	5.48	1.69	17.76	

Table S4. P-values for fear scale analyses in dogs and cats.

	**Cat**	**Dog**
Other procedures	0.969	-
Nail Clip Only	0.925	**0.036**
pre-visit meds	0.052	**0.015**

Table S5: Predicted values, odd ratios and 95% confidence intervals for factors associated with cat fear scale and procedures at the vet.

**CAT**		proportion showing fear	se	Odds Ratio	LCI	UCI	P value
Nail clip only?	No	0.622	0.046	-			0.925
	Yes	0.615	0.047	0.97	0.562	1.69	
Pre-visit pharmaceuticals	No	0.605	0.036	-			
	Yes - effective	0.625	0.099	1.09	0.452	2.61	0.152
	Yes - ineffective	1	-	-	-	-	
Other procedures	No	0.620	0.049				0.969
	Yes	0.617	0.045	0.99	0.684	1.85	

Table S6: Predicted values, odd ratios and 95% confidence intervals for factors associated with dog fear scale and procedures at the vet.

**DOG**		proportion showing fear	se	Odds Ratio	LCI	UCI	P value
Nail clip only?	No	0.738	0.039	-			0.036
	Yes	0.857	0.038	2.13	1.03	4.41	
Pre-visit pharmaceuticals	Yes - effective	0.571	0.110	-			
	No	0.794	0.031	2.89	1.129	7.41	0.015
	Yes - ineffective	0.944	0.054	12.75	1.421	14.40	

Table S7: Codes and frequencies for free text responses to the question “Has there ever been a dog you have not been able to clip the nails of while conscious (even if sedated or given pre-visit pharmaceuticals)?”, “If yes, did you suggest an alternative approach?” (n=189).

Code	Frequency	Example (respondent number)
Sedation or general anaesthesia to facilitate nail clipping	87	“returning fasted for proper sedation, ie medetomidine. we do not fight with animals in our clinic to clip nails, if the animal won’t allow the feet to be touched and nails clipped with out struggling alternative plans are made” (73)
Dispense pre visit pharmaceuticals	69	“Booking in with a vet to discuss pre-visit pharmaceuticals to make it a better experience for the animal” (78)
Desensitisation and or counterconditioning at home	27	“more desensitisation at home to improve response to touching feet” (62)
Recommend use of a scratch board or scratch pad at home to wear down nails	24	“I suggested a scratch board to help file down nails” (80)
Recommend wearing nails down on rough surfaces by walking on concrete or similar	17	“frequent walking on rough surface to file nails naturally” (208)
Postpone nail clip until the animal is having sedation or a general anaesthetic for another reason	15	“After discussion with DVM it was elected to proceed with nail trim under general anaesthetic whilst they had another procedure performed (COHAT) [comprehensive oral health assessment and treatment] - this just delayed the nail trim for a little bit” (185)
Recommend owner clips or files nails at home	14	“Depending on the dog (and owner), I'm fearful dogs I like to show owners how to do them, if they're willing to do them at home” (230)
Desensitisation or counterconditioning at the veterinary practice	12	“positive vet visits (no treatment, just cuddles and snacks)” (219)
Try again another day	6	“We made a new plan about different qty of pharmaceuticals, quieter timing within clinic and coming into the clinic via a different door. Next trip was very successful and dog was completely changed with this new plan” (244)

Other (trying different team members, different rooms/areas of the clinic, asking owner to hold differently, owner not happy with advice, not having a choice about alternatives, remove fearful dog from owner)	8	“some of our vets do not care and expect us to just 'get it done'” (196)
Incremental nail clipping	5	“had a patient that would come in once a week for 1 or two nails to be clipped because she was so anxious about getting them done - we took it very slow and respected when she had enough” (118)
Veterinary team members perform a home visit	4	“Home vet with pre visit sedation” (148)
Reconsider the need for nail clipping altogether	3	“To either come back on more pre visit pharmaceuticals or to not do them as they are not long enough to be causing any major issues and fighting the stressed patient isnt worth it” (26)

Table S8: Codes and frequencies for free text responses to the question “Has there ever been a cat you have not been able to clip the nails of while conscious (even if sedated or given pre-visit pharmaceuticals)?”, “If yes, did you suggest an alternative approach?” (n=129).

Code	Frequency	Example (respondent number)
Sedation or general anaesthesia to facilitate nail clipping	68	“Usually cats that are aggressive for anything at vets so heavy sedation/GA is required” (86)
Dispense pre visit pharmaceuticals	42	“try again with pre visit medication” (110)
Postpone nail clip until the animal is having sedation or a general anaesthetic for another reason	13	“postpone until next general anaesthetic with dental prophy”(93)
Recommend the use of scratching posts or scratch pads at home to wear nails down	11	“discussed scratching post and other husbandry options” (128)
Desensitisation and or counterconditioning at home	8	“desensitisation with O including treats” (154)
Recommend owner clips or files nails at home	8	“If cat was good at home, suggested owner attempt at home” (84)
Reconsider the need for nail clipping altogether	6	“again discussed the merit of pushing just for nails, opted to stop” (54)
Other (synthetic pheromones, nail caps, pain assessment and medical examination,	5	“pain assessment, medical exam +/- sedation” (104).

cooperative care training or description of cat's behaviour)		
Veterinary team members perform a home visit	3	"home visits for the cat" (243)
Try again another day	3	"Tried again another day where the cat hadn't had as much time to get worked up in carrier. Often successful" (244)
Modify animal handling next time	2	"less restraint on application" (249)
Educate owners regarding keeping cats calm during transport to the vet	2	"desensitisation training to cage/transport" (216)
Incremental nail clipping	2	"doing one at a time a day at home + teaching O how" (36)
Unsure	2	"Unsure"

Table S9: Codes and frequencies for free text responses to the question "If resources were not limited, how might you approach clipping an animal's nails?" (n=238)

Code	Frequency	Examples (respondent number)
Densensitisation and counterconditioning at the vet (including use of treats, toys)	119	"Desensitisation visits, include training on desensitisation in puppy classes" (131)<br><br>"Ensure cat is accustomed to area where you will perform the clip and that they are in a good mood. Give treats and play with them, then do clip. Give treats again and play time to finish off so they associate the procedure with good things" (163)
Nail clips should be performed under sedation general anaesthetic or with PVPs on board more frequently or always	110	"sedation/anxiolytics prior to appt regardless of how well the animal feels about getting their nails done" (119)<br><br>"All patients should be given sedation prior to the visit for the nail clipping. It should be mandatory at least for cats. Or we should not offer nail clipping unless the animal is being sedated and is unable to be done at a groomer etc." (187)
Resources to educate owners regarding nail management at home	83	"Education on training the pet to tolerate and the owner how to do it themselves" (150)<br><br>"comprehensive education of owner to do at home" (170)

More time to approach animal and nail clipping slowly	71	"Be able to spend more time giving the pet space when they become stressed rather than rushing" (43)<br><br>"Always allow ample time to get the patient comfortable with us in clinic, spend as long as it takes for that to happen" (70)
Clip nails in a quiet calm environment or dedicated space where animals are most comfortable	51	"Ideally separate consult areas for cats and dogs that are not surrounded by the hustle and bustle of the work space or other noisy patients/clients" (103)<br><br>"less clinical room. I.e. more of a calm homely room set up. relaxed space for owners to encourage calm behaviour with the pet" (128)
Multiple or more staff are required to perform this task adequately	44	"More people- easier to gently keep animal calm + easier to give treats + distract!" (166)<br><br>"For dogs have 2 nurses (1 to hold and 1 clipping) and owner or a 3rd nurse give treats etc." (206)
It is generally better for animals when their owners are present	36	"owners present to give lots of treats and reassurance" (46)<br><br>"Ideally the patient has their nails clipped with the owner present too. This allows easier and more open conversations if PVPs are required as well as client education on how to do nail trims themselves at home if they'd wish as well as how it can be made a better experience for their pet if needed" (185)
Less, more or different restraint methods	33	"safer restraint methods" (53)<br><br>"Quietly, calmly with minimal restraint. Work with the animal and see what type on restraint, if any is needed" (222)
Being able to stop when animals are stressed	24	"If stress is encountered say no immediately and find alternative not push through with them stressed" (65)<br><br>"Nails only as patient allows e.g if can only do one at a time then one at a time it is" (195)

Appropriate and well maintained equipment should be available	12	"sharp, size-appropriate nail clippers" (44)<br><br>"better nail clippers that are sharpened regularly" (136)
Prefer not to clip nails at all or only if medically necessary	11	"I would rather not do nail clips on conscious patients" (88)<br><br>"Ideally only done nail if it is therapeutic related (torn or embedded nails, diseased nails, quality of life related)" (122)
More staff training	8	"all staff fear free trained" (37)<br><br>"Low stress training for staff" (52)
Nail clipping strategies should meet individual patient needs	6	"Whatever suits the patient. No patient is the same and each have their own considerations, older patients may have arthritis so it may hurt them when manipulating feet to get trimmed, younger patients may have anxiety and need pre-visit pharmaceuticals, and some patients may be stressed or less stressed with their owner so flexibility to be able to take them to a different area or keep them with their owner. Pretty much to have the resources to be able to provide exactly what the patient needs to be comfortable during a nail trim" (78)<br><br>"It is a matter of working with each animal as an individual and finding what works for them" (139)
Animals should be separated from their owners during nail clips	5	"Away from owner in most situations" (126)<br><br>"They would be brought through immediately away from the owner- being near the owner causes them to feed off each others stress and the owners tend to be really forceful" (177)
We do a good job already or no change	5	"Probably 80% of the nails I clip are easily done with minimal restraint. You need to be careful and not cut too short. I certainly don't think every patient should be drugged unless it is warranted" (63)

		"I wouldn't approach it differently to how I do now, as a team we always ensure theirs time and staff available to help one another. never over booking" (110)
Home visits	2	"Home visits at specific times in the day (determined by pet routine)" (243)
Not sure	2	"Unsure" (203)
Invest in research and development	1	"I would also spend a few billion investing in a reversible NON alpha-2 sedatve agent. OR a vaccine that prevents nails from overgrowing at all" (85)

Table S10: Codes and frequencies for free text responses to the question "Is there anything else you want to add?" (n=68).

Code	Frequency	Examples (respondent number)
Nail clips are undervalued by clients or veterinary team members	21	"Nail clipping is one of the most undervalued services we provide. Owners do not appreciate how many staff members it can involve to do it well" (85)<br><br>"Nail trims are one of the hardest things I have to do in the clinic, they are stressful on both me and the animal, risky in terms of scratches, bites and poor posture. Clients do not see the value either" (158)
There is a need for more training of veterinary team members regarding nail clipping and minimising patient fear, anxiety, and stress	19	"having the same protocol across clinics in the same region would be great as in our area one clinic will pin and hold the animals down and just muzzle then they come to us and we won't and get complaints and abused. Vets also need a better understanding and respect for us nurses, if we say we cannot do the nails then we cannot do them, it's not because we are lazy it's because they are stressed or aggressive and we do not wish to make it worse or get injured" (196)<br><br>"a deeper understanding of body language in animals would help make this procedure more agreeable for all parties. the root cause of nail clips being the horrendous task they are is a lack

		of proactive training and education on all fronts" (87)
We should not be performing nail clipping as frequently, or at all, in veterinary settings	14	"It's odd how often people bring their pets in to have their nails trimmed. Surely there are some at home alternatives owners could be trying and the whole clinic can get on board with recommending them. I think there is way too much stress in nail trimming for what the outcome is" (18)<br><br>"We have to preform so many other treatments on pets which cause stress so unless it is directly affecting their health (or it is during a period of time when they are sedated ie during surgery prep) nail clips should be left to groomers in a grooming setting. We don't go to our gp to have our nails trimmed why should pets be any different" (201)
Approaches for minimising fear anxiety and stress during nail clipping	14	"More training while animals are young prior to needing nail clips as they get older" (22)<br><br>"I personally think vet staff should only perform nail trims with oral sedation, in-clinic sedation, during procedures, etc but not as a standard service like groomers do" (136)
There is pressure to get the job done	9	"Persevering to trim nails because of client or employer pressure to just get it done is a huge OHS [Occupational Health and Safety] risk and is unsafe for both employee and the animal"(75)<br><br>"Clients still do not understand or accept fear free practices. They just want the animals held down abs the nails done. We have had many complaints when we say we should stop and try other options" (181)
There should be more time to take breaks, clip nails incrementally and to take individual animal needs into account	9	"it is OK to not perform and push through a nail clip if it is in the animal's best interests to stop" (45)

		“I dont rush and I always try to find the perfect way to hold to keep the animal comfortable as they are all different and prefer different ways of handling” (250)
Comments about the survey itself	7	“This survey did not allow space to discuss usual or preferred practice, unfortunately my last dog nail clip was not a good experience, however after 3 failed attempts we as a team agreed to cancel the attempt and to re-asses our future approach. I feel this was a win for the patient and a vast improvement from the physical pining down I was taught when I first joined the industry nearly 20years ago” (71)<br><br>“it honestly builds so much trauma for animals in vet clinics... our regular nail trim patients don't "get used to it" they either shut down or get worse .... it also creates a lot of compassion fatigue for us glad someone is doing this study!” (199)
Feelings regarding nail clipping in general or particular experiences	7	“Hate nail clips...if there was one job I would refuse to do it would be clipping dog nails (cats I don't mind)” (106)<br><br>“I hate nail clips when I have to wrestle with animals!” (144)
Need for owners to be educated in low stress and fear free approaches	4	“Client education would be really helpful - not asking to "just clip them short", owners being more willing to put time into desensitisation, owners happy to come back with PVPs on board or considering if the nail clip is really necessary if their pet is very stressed” (48)<br><br>“Clients at my clinic appear to have a fear of giving mild sedative medication to their pets prior to consultations. Maybe more time needs to be allocated to working with the owners on behaviour management/ training

		to minimise fear of these medications?" (103)
